# Circulating Vascular Progenitor Cells and Central Arterial Stiffness in Polycystic Ovary Syndrome

**DOI:** 10.1371/journal.pone.0020317

**Published:** 2011-05-31

**Authors:** Cecile Dessapt-Baradez, Maria Reza, Ghayathri Sivakumar, Maria Hernandez-Fuentes, Kostas Markakis, Luigi Gnudi, Janaka Karalliedde

**Affiliations:** 1 Cardiovascular Division at Guy's and St Thomas and King's College Hospitals, King's College London, London, United Kingdom; 2 Immunology, Infection & Inflammatory Disease Division and NIHR Biomedical Centre at Guy's and St Thomas and King's College Hospitals, King's College, London, United Kingdom; Leiden University Medical Center, Netherlands

## Abstract

**Objective:**

Subjects with Polycystic ovarian syndrome (PCOS) are at increased risk of Type 2 diabetes mellitus (T2DM). The mechanism of this enhanced risk is unclear. Circulating vascular progenitor cells (VPC) are immature bone marrow derived cells capable of differentiating into mature endothelial cells. VPC number/function and central arterial stiffness predict cardio-metabolic disease in at-risk populations.

**Design:**

We studied VPC and arterial stiffness measures in non-obese PCOS subjects as compared to age and body mass index (BMI) matched healthy controls in a cross–sectional study.

**Methods:**

Fourteen subjects with PCOS and 12 controls of similar age, BMI (all <30 kg/m^2^) and metabolic profile were studied. VPC number and *in vitro* function were studied by flow cytometry and tube formation assays respectively. Augmentation index (AIx), a measure of central arterial stiffness, and central (aortic) blood pressures (BP) were measured by applanation tonometry.

**Results:**

Subjects with PCOS had a reduced number, mean±SEM, of circulating CD34^+^133^+^ VPCs (317.5±51.0 vs. 558.3±101.2, p = 0.03) and impaired *in vitro* tube formation (completed tube area 1.0±0.06 vs. 1.2±0.05×10^6^ µm^2^ p = 0.02). PCOS subjects had significantly higher AIx (18.4±1.9% vs. 4.9±2.0%) and this difference remained significant even after adjustments for age, BMI and smoking (p = 0.003) in multivariate analyses. Central systolic and pulse pressure were higher in PCOS subjects but these differences were not statistically significant after adjustment for age. Brachial systolic and pulse pressures were similar. VPC number/function and arterial stiffness or BP measures were not correlated.

**Conclusions:**

Non-obese PCOS is characterized by a reduced VPC number, impaired VPC function and increased central arterial stiffness. These changes in novel vascular risk markers may explain the enhanced risk of T2DM and CVD in PCOS.

## Introduction

The diagnosis of polycystic ovarian syndrome (PCOS) confers a higher risk of type 2 diabetes mellitus (T2DM) and possibly cardiovascular disease (CVD) which often becomes more pronounced as subjects age (1). The pathophysiology of the enhanced cardio-metabolic risk in PCOS is not fully explained by traditional risk factors [1]. CVD and T2DM may share a common pathophysiology as proposed in the ‘common soil’ hypothesis with data indicating that both are vascular conditions [2]. Circulating vascular progenitor cells (VPC) are a subset of mononuclear cells derived from the bone marrow [3] that retain the ability to proliferate and differentiate into mature endothelial cells which contribute to vessel homeostasis and repair. Of interest progressive deficit in VPC number/function contributes to the development of atherosclerosis and associates with impaired glucose metabolism including T2DM [4]. Central arterial stiffness, a hallmark of vascular ageing, is an independent predictor of CVD and associates with risk of T2DM [5]–[7]. Augmentation index (AIx) is a validated measure of central arterial stiffness and central aortic pressure changes predict adverse CVD outcomes in at risk populations independent of brachial blood pressure [6],[7]. Obesity *per se* reduces VPC number/function, increases arterial stiffness and enhances risk of CVD and T2DM in PCOS [1],[7]–[9].

Whether there are differences in VPC and central aortic pressures, two emerging vascular bio-markers of cardio-metabolic risk, in non-obese PCOS subjects is unknown.

## Results

Fourteen subjects with PCOS and 12 healthy controls were studied. More than 2/3rds of subjects in each group were Caucasian and the number of smokers (n = 2 in PCOS and n = 1 control) was similar. The clinical and biochemical characteristics' of PCOS subjects and healthy controls are shown in Table 1. There was no statistically significant difference between the PCOS vs. control group, in age, weight, BMI and waist circumference (Table 1). All patients had a BMI<30 kg/m^2^ and were normotensive [brachial systolic (SBP) and diastolic blood pressure (DBP) <130/90 mmHg respectively]. Total cholesterol, mean±standard error of the mean, (4.6±0.2 vs. 4.0±0.1 mmol/l p = 0.055), plasma triglycerides (1.0±0.1 vs. 0.7±0.1 mmol/l p = 0.055) were higher in the PCOS group. Serum total testosterone was 27% higher in PCOS but this difference did not reach conventional statistical significance (2.6±0.3 vs. 1.9±0.2 p = 0.06). Fasting plasma glucose (4.7±0.1 vs. 4.5±0.1 mmol/l p = 0.2) did not differ between the two groups. Insulin resistance estimated by HOMA was higher (nearly 36%) in the PCOS group (2.5±0.4 vs. control 1.6±0.3) but this difference was not significant (p = 0.2). Of note all subjects had fasting plasma glucose <6.0 mmol/l.

**Table 1:** Selected clinical, biochemical and brachial blood pressure measurements in non-obese subjects with PCOS and age and body mass index matched healthy controls.

	PCOS	Controls	P value
	n = 14	n = 12	
**Age (years)**	26.4±1.0	23.2±1.5	0.08
**Body mass index (Kg/m^2^)**	24.2±0.8	23.0±0.7	0.26
**Weight (kg)**	65.9±3.1	61.0±1.2	0.21
**Waist circumference (cm)**	86.3±2.5	82.1±1.8	0.22
**Brachial systolic blood pressure (mmHg)**	115.2±3.4	108.7±3.0	0.17
**Brachial diastolic blood pressure (mmHg)**	76.2±2.6	68.1±2.6	0.05
**Brachial pulse pressure (mmHg)**	38.9±1.0	40.0±1.8	0.60
**Brachial mean arterial pressure (mmHg)**	89.2±2.9	82.0±2.6	0.08
**Augmentation Index % corrected for heart rate of 75 beats per minute**	18.4±1.9	4.9±2.0	0.001*

Data are presented as mean±SEM.

PCOS subjects had an almost a three fold higher AIx. Mean brachial arterial blood pressure (a measure of the passive distension of the arterial wall), brachial SBP and pulse pressures were not elevated and similar in both groups (Table 1).

As age and smoking can affect circulatory parameters, univariate correlations of AIx, central arterial pressures and CD34+CD133+ VPC number with PCOS diagnosis, age and smoking were performed (Table 2). These analyses showed a statistically significant correlation between AIx and PCOS and between AIx and age. There was no significant association between AIx and smoking. Similar results were observed for central aortic SBP and pulse pressure (Table 2). In univariate analyses BMI was not significantly associated with AIx, central aortic blood pressures and VPC number or function.

**Table 2:** Univariate correlations of AIx, central arterial pressures, and CD34+CD133+ VPC number with PCOS diagnosis, age and smoking.

Augmentation Index as dependent variable	Coefficient of correlation r value	Significance p value
PCOS	0.65	0.01*
Age	0.60	0.02*
Smoking	0.31	0.13

Central aortic systolic blood pressure as the dependent variable		
PCOS	0.45	0.03*
Age	0.42	0.04*
Smoking	0.27	0.20

Central aortic pulse pressure as the dependent variable		
PCOS	0.46	0.02*
Age	0.44	0.03*
Smoking	0.38	0.06

CD34+CD133+ VPC number as the dependent variable		
PCOS	−0.50	0.01*
Age	−0.28	0.20
Smoking	−0.30	0.14

In a multivariate regression analysis model with AIx as the dependent variable, PCOS emerged as the most important factor followed by age (Table 3). The results of these analyses show that PCOS subjects independent of age, BMI and smoking status have significantly greater central arterial stiffness as determined by AIx. The results of the model suggests that PCOS subjects have nearly 11% greater AIx than controls independent of age which is equivalent to almost 10 years of vascular ageing [10]. The adjusted R^2^ value from this model suggests that 55% of the variance of AIx is predicted by PCOS and age. In independent samples (unpaired) t-tests analyses, PCOS subjects had higher central aortic systolic blood pressure (103.7±2.4 vs. 94.9±2.2 mmHg p = 0.03), central aortic diastolic blood pressure (75.6±1.8 vs. 69.7±2.4 mmHg p = 0.08), central aortic pulse pressure (28.2±1.0 vs. 25.1±1.1 p = 0.04). However these differences were not statistically significant after adjustment for age. When central aortic SBP was entered as the dependent variable, the multivariate regression model results indicated that PCOS independent of age, BMI and smoking does have some effect albeit not reaching conventional statistical significance (PCOS unstandardized coefficient B 8.23, p value = 0.09. We did not observe a statistically significant effect of PCOS, independent of age, BMI and smoking, on central aortic pulse pressure when the latter was entered as the dependent variable in a multivariate regression model.

**Table 3:** Age and BMI independent effects of PCOS on central arterial stiffness, as measured by augmentation index.

Variable	Unstandardized Coefficient B	Significance p value
PCOS	11.04	0.003
Age	0.86	0.015
BMI	0.08	0.885

Subjects were 12 healthy controls and 14 subjects with PCOS matched for body mass index.

**Figure 1:**
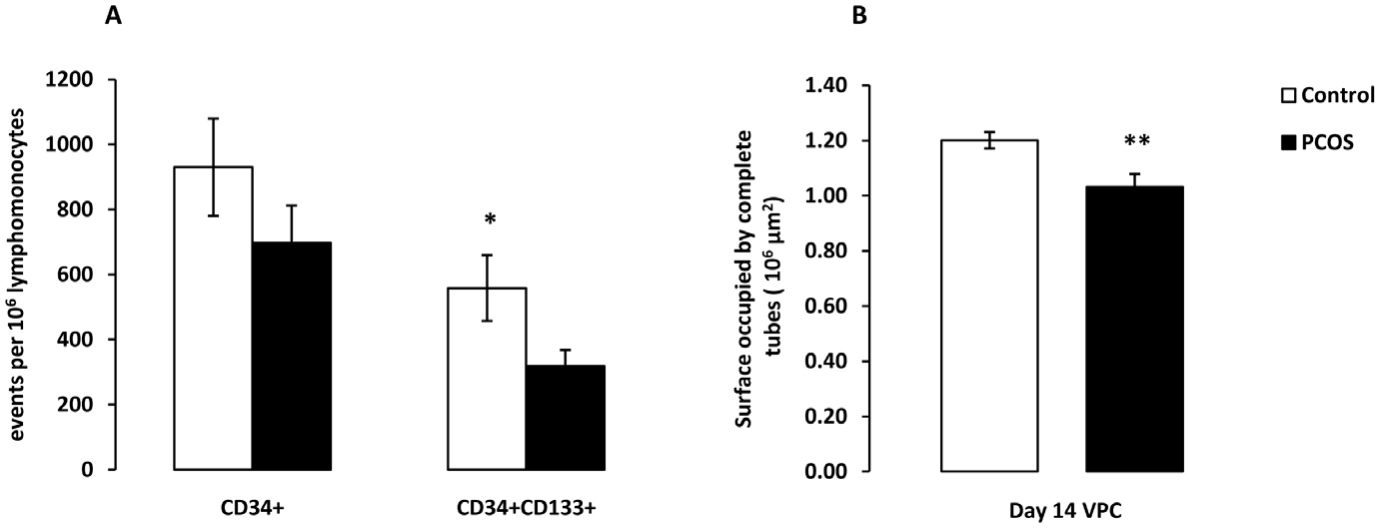
Circulating vascular progenitor cell (VPC) number (A) and in-vitro function (B) in healthy controls (□) and PCOS (▪) subjects. Circulating CD34^+^/CD133^+^ VPC number is reduced (A) and VPC function impaired (B) in PCOS patients *vs*. healthy control. (* p = 0.03, ** p = 0.02). Data are presented as mean±SEM. For A, PCOS n = 14 vs. control n = 12, for B PCOS n = 10 vs. control n = 8.

**Figure 2:**
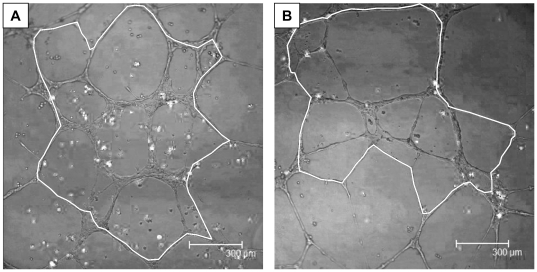
Tubule formation assay in healthy controls (A) and PCOS (B) patients. Panels A and B are representative pictures of the tubule network formed by the VPC (white dots) and HUVECs. The white line represents the surface area which was measured i.e. area formed by complete closed tubes.

Subjects with PCOS had a significantly lower number of circulating CD34^+^133^+^ VPC, 317.5±51.0 vs. 558.3±101.2, per 10^6^ lymphomonocyte events p = 0.03 (Figure 1a). PCOS subjects also had lower number CD34^+^ cells (697.5±114.5 vs. 929.9±149.3p = 0.2). CD34^+^CD133^+^KDR^+^ and CD34^+^KDR^+^ cells per 10^6^ lymphomonocyte events were very small in both groups with no statistically significant differences noted between PCOS subjects and healthy controls. Age and smoking status were not significantly associated with CD34+CD133+ VPC number in univariate correlation analyses (Table 2). VPC functional assays were performed in 10 PCOS and 8 control consecutive patients from the population described comparable for all characteristics. The phenotype of VPC cultured for 7 days and 14 days did not differ between the groups however day 14 VPC were characterised by greater expression of endothelial markers such as von Willebrand factor, CD31 and eNOS compared to day 7 VPC which expressed predominantly white cell markers such as CD14 as previously reported [11]. Migration and tube formation assays with VPC cultured for 7 days showed similar results in both groups (data not shown). VPC cultured for 14 days from PCOS subjects had less migration (22,553±589 vs. 25,062±1523) but this difference did not reach statistical significance (p = 0.23). However VPC cultured for 14 days from PCOS subjects showed a significantly reduced completed tube area (1.0±0.06 vs. 1.2±0.05×10^6^ µm^2^ p = 0.02) with VEGF-A compared to controls (Figure 1b and Figure 2).

## Discussion

This is the first study demonstrating a lower circulating number and impaired function of VPC in PCOS. PCOS subjects had significantly lower number of CD34^+^133^+^ cells, which are early VPC capable of differentiating into endothelial cells in the setting of vascular inflammation or injury, and are central to repair of damaged endothelia [12],[13]. We also report, after adjustment for age and BMI, significantly greater central arterial stiffness in non-obese PCOS subjects as compared to matched controls despite similar brachial and central blood pressures a finding not previously demonstrated. Our results suggest that PCOS subjects independent of age and smoking status have almost a 10 year increase in vascular ageing [10].

In-vitro measures of VPC function in PCOS have not previously been reported. Tube formation was significantly reduced only in VPC cultured for 14 days from PCOS subjects suggesting impaired vascular repair. The characteristics/phenotype of cells cultured for 7 and 14 days did not differ between PCOS and controls, however VPC cultured for 14 days expressed predominantly endothelial markers and this may explain the VPC function results observed [11].

Our results may explain at least in part the pathophysiological mechanism of endothelial dysfunction in PCOS which is not fully explained by traditional cardio-metabolic risk factors [14]. In contrast to our findings, a recent study demonstrated no difference in CD34^+^133^+^ cell number between PCOS and controls. In this study subjects were older, included obese cases and controls and subjects with PCOS were not selected on the basis of menstrual irregularity [15]. In a recent large cross-sectional study of 425 subjects categorised according to carbohydrate metabolism status, a reduction in the number of VPC marked the clinical onset of T2DM, independent of other known risk factors [4]. The authors of this study proposed that defective mobilisation and increased apoptosis of VPC may account for these results [4] although the exact mechanism(s) by which reductions in the number and function of VPC result in an increased risk of T2DM remains unclear. The number of CD34^+^133^+^ cells has been found to be reduced in patients with essential hypertension when compared to normotensive individuals [16]. However we did not observe any correlation between brachial and central arterial pressures and VPC numbers or function which suggests that the changes in VPC and arterial stiffness are potentially independently associated with the ‘enhanced vascular risk milieu’ that characterises PCOS. VPC number has been reported to vary depending on timing of menstrual cycles, but the literature is discordant in that some authors report higher VPC numbers in the follicular phase whilst others report higher numbers in the luteal phase [17],[18]. Moreover Fadini *et al* did not observe difference between follicular and luteal phases of the cycle but did note an increase in VPC during ovulation [19]. Lemieux *et al* have also reported a similar pre-ovulatory surge in VPC numbers but no difference in numbers between menstrual and mid luteal phases of the menstrual cycle [20]. Interestingly VPC function does not appear to be affected by the timing/phase of the menstrual cycle [17].

Recent evidence is that central arterial stiffness and blood pressures may be more clinically relevant than peripheral (brachial) pressures for the pathogenesis of CVD and T2DM [6],[7]. Rajendran *et al* demonstrated a 4 fold higher AIx in obese PCOS subjects compared to controls but no significant difference between lean PCOS (BMI<25 kg/m^2^) and controls [15]. In our study, waist circumference, a clinically proven measure of central obesity and associated cardiovascular risk, was not significantly different between control and PCOS subjects. This suggests that central obesity is unlikely to be the most important cause of the increased arterial stiffness observed in PCOS. The literature in this area is conflicting with some studies reporting that central obesity, rather than PCOS per se, is associated with increased arterial stiffness and others reporting that obese and non-obese PCOS subjects have greater arterial stiffness compared to matched controls [9],[21],[22]. Soares *et al* reported increases in arterial stiffness in PCOS in the absence of obesity and hypertension in non-obese PCOS (mean BMI 22.7) subjects as compared to matched healthy controls [21]. In contrast there are reports that non-obese PCOS subjects do not have greater arterial stiffness as compared to matched controls [15],[23]. Kelly et al in their seminal paper in this field reported that obese PCOS subjects (mean BMI 33.2) had greater arterial stiffness that controls with similar BMI and waist hip ratio [22].

Vascular ageing is characterized by the loss of elasticity in the wall of the aorta and as a consequence there is increased central arterial (aortic) stiffness. This results in the faster return of pressure waves from distal sites (resistance vessels) where impedance mismatch occurs, which shifts augmentation of blood pressure from diastole to systole resulting in elevated central SBP and AIx. The value of arterial stiffness as a therapeutic target in PCOS was recently demonstrated by the reduction in AIx, brachial and central blood pressures with metformin [24]. Our study was cross-sectional proof of concept study and therefore larger prospective studies are required to clarify if the differences in arterial stiffness, VPC number and function are a cause or an effect of PCOS and to determine the long term consequences of these changes. In conclusion, we have demonstrated the novel findings of reduced number and function of VPC, raised central aortic blood pressures and arterial stiffness despite similar ‘normal’ brachial blood pressures in PCOS subjects compared to matched healthy controls. Our results highlight the need for larger confirmatory studies to evaluate if these differences in emerging predictors of cardio-metabolic disease explain, at least in part, the enhanced risk of T2DM and CVD in PCOS.

## Materials and Methods

### Ethics statement

The study was approved by the research ethics committee of Guy's and St. Thomas' Hospitals and was undertaken in adherence to the Declaration of Helsinki. Written consent was obtained from all patients who participated to this study.

Inclusion/exclusion criteria and subject characteristics: Subjects with a confirmed diagnosis of PCOS by the Rotterdam Criteria [25] who were not on any medication and attending the endocrine clinics at Guy's and St Thomas' Hospital London were studied. All PCOS subjects had a history of irregular/infrequent menstrual cycles and polycystic ovaries, as defined by ultrasonography (the presence of 12 or more follicles in each ovary measuring 2 to 9 mm in diameter, and/or ovarian volume >10 mL). Furthermore all PCOS subjects had a clinical history and clinical signs of hyperandrogenaemia (hirsuitism and acne) that fulfilled the Rotterdam diagnostic criteria. The participants of the consensus group the defined the Rotterdam criteria suggest that the primary clinical indicator of androgen excess is the presence of hirsuitism and that the presence of acne is a marker for hyperandrogenism particularly in patients with irregular menses [25]. A limitation of our study was that hirsuitism was not scored by the Ferriman-Gallwey score.

Exclusion criteria included a history of T2DM, other endocrine illnesses (e.g.thyroid disease, adrenal disease, pituitary disease), CVD and hypertension. All controls were healthy with no clinical or biochemical features/characteristics of PCOS and had regular menstrual cycles. Control subjects were not on any medications and had measurements performed in the follicular phase of the menstrual cycle.

Central aortic blood pressure and AIx were measured by applanation tonometry (Millar tonometer, Millar Instruments, Houston, TX) using the Sphygmocor system (Atcor, Sydney, Australia) at the radial artery as previously described [26]. AIx is calculated as the ratio of the augmentation pressure (AP) that results in the amplification of peak systolic blood pressure over the pulse pressure (PP) (AIx = AP/PP) and corrected for heart rate of 75 beats per minute [6],[7],[26]. Brachial blood pressure was measured in the dominant arm with the subject seated after a five-minute rest using an automated sphygmomanometer (Dinamap-8100T, GE-Medical, Slough, UK). The mean of three consecutive measurements was used for AIx, brachial and central blood pressure measures. Weight, height and waist circumference were measured using standard methodology as previously described [26]. Total cholesterol, plasma glucose as previously described [26], serum testosterone (competitive electro-chemiluminescent immunoassay, Testosterone II cobas^R^ Roche E170, Roche Diagnostics GmbH, Mannheim, Germany) and insulin (electro-chemiluminescent immunoassay Insulin cobas^R^ Roche Elecsys2010 Roche Diagnostics GmbH, Mannheim, Germany) were measured after an overnight fast. Insulin resistance was estimated using the homeostasis model assessment of insulin resistance (HOMA-IR) and calculated as fasting insulin x fasting glucose/22.5 [27]. T2DM and impaired fasting glycaemia was excluded by fasting plasma glucose levels <7 mmol/l and <6 mmol/l respectively and no previous history of symptoms of hyperglycemia and or casual plasma glucose ≥11.1 mmol/l [28].

Circulating VPC were isolated and cultured as follows. Mononuclear cells (MNCs) were isolated from 40 ml peripheral blood from healthy or PCOS subjects by density gradient centrifugation with Ficoll Paque PLUS (density 1.077 g/ml; GE Healthcare) according to manufacturer recommendations. MNCs (10×10^6^) were plated in 2 ml endothelial growth medium (EGM-2 MV bulletkit; Lonza), on fibronectin-coated six-well plates at 37°C in a 5% CO_2_ humidified incubator. Under daily observation, after 4 days of culturing, medium were changed and non-adherent cells removed. Adherent VPC at 7 days of culture appear elongated and spindle shaped. Thereafter, medium were replaced every 3 days, and cell differentiation followed for 14 days in total.

Circulating VPC were characterized and counted by flow cytometry and immunofluorescence as previously described [11]. Briefly, leukocytes were isolated from peripheral blood after red cells lysis with ammonium chloride buffer. Following Fc receptor blocking (FC blocker - Miltenyl Biotec, Bergisch Gladbach, Germany) cells were labelled with CD133-PE, CD34-FITC (Miltenyl Biotec, Bergisch Gladbach, Germany) and VEGFR-APC (R&D System, Abondgon, UK) antibodies on ice in the dark following manufacturer instructions. Cells were fixed using BD lysing solution and acquired using a flow cytometer- the FACS Calibur analyser (FACS Calibur; BD Sciences, Franklin Lakes, NJ, USA). At least 500 CD34^+^133^+^ events were collected for each subject and typically showed more than 1×10^6^ events in the lymphomonocytes gated area.

Prior to performing functional assays, circulating VPC from healthy and PCOS subjects were characterised by immunofluorescence as previously described [11]. Human umbilical vascular endothelial cells and macrophage line U937 were used as positive control for endothelial and monocytic markers respectively, negative control was included by omitting the first antiserum. Non-specific Fc receptors were blocked using blocking buffer (CAS block, Zymed laboratories-Invitrogen, Paisley, UK). Primary antisera (Santa Cruz Biotechnology, SantaCruz, CA, USA) and secondary AlexaFluor488 or Texas Red antisera were used at 1/50 dilution (ChemMate™ Antibody Diluent, Dako, Ely, UK). Cells were stained in duplicate, and four different fields (∼20–30 cells) were assessed by a blinded investigator. At 7 days culture, cells expressed mainly VEGFR2, eNOS, CD45 and CD14. At 14 days culture, cells expressed more endothelial specific markers (VEGFR2, CD144, Von Willebrand Factor-VWF, CD31, and eNOS) while a reduction in cells positive for CD14 was observed as previously described [11]. Most cells expressed CD45 at 14 days in both groups. Stem cell markers CD34 and CD133 were positive in a small proportion of cells.

Circulating VPC functional assays were performed as previously described in 10 PCOS subject and 8 controls using 7-days and 14-days cultured VPC in the presence and absence of Vascular Endothelial Growth Factor (VEGF)-A [11], [12].

### Statistical analyses

All measurements were performed blinded to group allocation. The primary aim/endpoint of the study was to evaluate if there were differences in VPC number between non-obese PCOS subjects and age and BMI matched healthy controls. As this was a proof of concept study no formal power calculation was performed. Shapiro-Wilk test was used to test if the distribution of the variable was normal. HOMA-IR, CD34^+^ cells, CD34^+^133^+^ cell numbers were skewed, and log transformation of data resulted in a normal distribution. Comparisons between PCOS and control group of variables which were normally distributed was made using the unpaired (independent samples) Student's t-test. CD34^+^CD133^+^KDR^+^ and CD34^+^KDR^+^ cell counts were not normally distributed and differences between groups were tested by the Kruskall-Wallis test. As age, BMI and smoking may affect circulatory parameters, univariate regression analysis appropriate for the normal (Pearson's correlation) or not normal distribution (Spearman's correlation) of the variable was performed to test for associations of AIx, central arterial pressures and CD34+CD133+ VPC number with smoking, age, BMI and PCOS diagnosis.

Correlations which were significant at less than the 10% level were entered in a multiple regression model with AIx, central aortic systolic blood pressure and central aortic pulse pressure as the dependent variable, to determine which variables independently best predicted circulatory parameters. All analyses were performed with SPPS version 15.0. p<0.05 was taken as indicating statistical significance, and all tests were 2-tailed.
